# Risk-based inspection as a cost-effective strategy to reduce human exposure to cysticerci of *Taenia saginata* in low-prevalence settings

**DOI:** 10.1186/s13071-018-2839-z

**Published:** 2018-04-19

**Authors:** Bhagyalakshmi Chengat Prakashbabu, Laura Rebecca Marshall, Matteo Crotta, William Gilbert, Jade Cherry Johnson, Lis Alban, Javier Guitian

**Affiliations:** 1 Royal Veterinary College, Hatfield, AL9 7TA UK; 2 Royal Society of Biology, Charles Darwin House, 12 Roger Street, WC1N 2JU, London, UK; 3 Danish Agriculture & Food Council, Axelborg, Axeltorv 3, DK-1609, København V, Copenhagen, Denmark

**Keywords:** *Taenia saginata* cysticercus, *Taenia saginata*, Cattle, Meat inspection, Risk-based inspection, Cost-effectiveness, ICERs

## Abstract

**Background:**

*Taenia saginata* cysticercus is the larval stage of the zoonotic parasite *Taenia saginata*, with a life-cycle involving both cattle and humans. The public health impact is considered low. The current surveillance system, based on post-mortem inspection of carcasses has low sensitivity and leads to considerable economic burden. Therefore, in the interests of public health and food production efficiency, this study aims to explore the potential of risk-based and cost-effective meat inspection activities for the detection and control of *T. saginata* cysticercus in low prevalence settings.

**Methods:**

Building on the findings of a study on risk factors for *T. saginata* cysticercus infection in cattle in Great Britain, we simulated scenarios using a stochastic scenario tree model, where animals are allocated to different risk categories based on their age, sex and movement history. These animals underwent different types of meat inspection (alternative or current) depending on their risk category. Expert elicitation was conducted to assess feasibility of scenarios and provide data for economic analysis. The cost-effectiveness of these scenarios was calculated as an incremental cost-effectiveness ratio, using the number of infected carcasses detected as the technical outcome.

**Results:**

Targeting the high-risk population with more incisions into the heart while abandoning incisions into the masseter muscles was found to reduce the total number of inspections and cost, while simultaneously increasing the number of infected carcasses found.

**Conclusions:**

The results suggest that, under reasonable assumptions regarding potential improvements to current inspection methods, a more efficient and sensitive meat inspection system could be used on animals categorised according to their risk of harbouring *T. saginata* cysticercus at slaughter. Such a system could reduce associated cost to the beef industry and lower microbial contamination of beef products, improving public health outcomes.

## Background

*Taenia saginata* cysticercus is found in cattle and is the larval stage of the adult tapeworm *T. saginata*. Cattle are infected through ingestion of feed or water contaminated with human faeces containing the eggs of the tapeworm. Eggs hatch into oncospheres, which penetrate the intestinal mucosa and reach internal organs and muscles *via* circulation. Oncospheres develop into cysticerci, becoming infective to humans in 10 weeks [1]. Humans become infected through ingestion of viable cysticerci in under-cooked or raw beef. The adult form of the tapeworm then develops in the human intestine. The infection in humans is usually asymptomatic but sometimes manifests with minor symptoms such as abdominal pain and anal pruritis. Hence, the disease is considered to have a low public health impact [2], and cases may go unnoticed and unreported. EU Directive 2003/99 recommends reporting *T. saginata* cysticercus cases within EU member states to the European Food Safety Authority (EFSA), yet very few countries report the data annually as it is not obligatory to do so [3]. A recently published literature review concludes that it is difficult to assess the public health relevance of *T. saginata* cysticercus due to the lack of an accurate estimate for prevalence in Europe [4].

*Taenia saginata* is globally distributed, with higher prevalence in countries with poor sewage management, which do not prevent the contamination of farm land with human faeces. The national prevalence estimates of European countries vary from as low as 0.01 to 6.2% [2, 4, 5]. The apparent prevalence of cysticercosis in the United Kingdom (UK), as calculated using the number of cattle with cysts detected at meat inspection for 2013–2015, is 0.013% [6]. Cysts can be viable (non-calcified) and non-viable (calcified). The predilection sites of *T. saginata* cysticercus are: heart, masseter, tongue, diaphragm and lungs. Currently in Europe, according to EU Regulation 854/2004, all bovine carcasses above six weeks of age are inspected for *T. saginata* cysticercus cysts as following: visual inspection and palpation of tongue, two deep incisions in the external masseter muscle and one deep incision in the internal masseter muscle, visual examination of the heart followed by lengthwise incision to open ventricles and cut through the interventricular septum and visual examination of diaphragm and oesophagus. The current regulation requires the condemnation of the whole carcass if there is generalised infection, defined as having more than one predilection site infected. In the case of localised infection (cysts in one predilection site), the affected part is removed and the carcass is kept in cold storage at temperature not exceeding -7 °C for a minimum of three weeks or at a temperature not exceeding -10 °C for a minimum of two weeks [7].

In the UK, data collected through voluntary laboratory testing shows there were 31 confirmed human cases of *T. saginata* in 2014. The source of these infections remains unknown and *Taenia *sp. is not part of the routine surveillance hence the data may not be representative of human infections (Dilys Morgan, DM, Public Health England, personal communication). It has been surmised that some such human infections in the UK may be acquired overseas. The estimates of prevalence in humans in Europe cited elsewhere, ranging from < 0.01 to 10%, are inferred from the sale of anti-parasitic drugs, or from a selected population such as hospitalised patients and a category of workers [1, 8, 9]. Despite its low public health impact, the presence of cysts in cattle leads to significant economic losses to the European meat industry and cattle farmers, due to degrading and condemnation of infected carcasses, increased processing costs and costs relating to meat inspection [10].

The current meat inspection technique is considered to have low sensitivity, with estimates ranging from as low as 10–50% depending upon the level of infection, with higher sensitivity in heavily infected carcasses. The sensitivity is assumed to be particularly low in low-prevalence countries such as the UK and Belgium where heavily infected carcasses are uncommonly found at meat inspection. A study carried out in Belgium estimated that the true seroprevalence of infection in cattle was 10 times higher than the apparent prevalence calculated through meat inspection [11]. The sensitivity of detection depends upon the level of development of cysts and the expertise of the meat inspector [12]. In addition to examining a large number of animals, the current method also involves incising valued cuts such as the internal and external masseters, leading to an increase in losses. Hence, presently a large amount of money is spent on an inspection method which provides limited public health protection by removing from the food chain only a small fraction of infected carcasses.

As developing and implementing new laboratory tests to increase sensitivity may not be cost-effective and practical, a recent shift has been to propose the development of risk-based inspection informed by our understanding of the epidemiology of infection in cattle [13–15]. The guidelines published by Codex Alimentarius now recommend evaluation of epidemiological data to inform risk-based surveillance for cysticercosis in domestic cattle [16]. Some studies have looked into various methods of risk-based surveillance. A common finding among them was the potential for age and sex as indicators of *a priori* risk of cyst presence [17, 18]. Studies conducted in Switzerland and Belgium showed an increase in sensitivity through more detailed meat inspection involving increased cuts into heart [9, 19, 20]. There are previous examples of launching a risk-based surveillance system for low prevalence zoonotic parasites. In Denmark, abattoirs that sell meat on the EU or national market are allowed by the EU to target inspections for *Trichinella* spp. on pigs which are considered high-risk due to being housed under non-controlled housing conditions [21]. In Canada, it is required to conduct an enhanced inspection, by incising forequarters and rounds of bovine carcass if the animal was known to originate from a *T. saginata* cysticercus infected herd [22].

In the UK, a recent study identified sex and age as risk factors for detection of *T. saginata* cysticercus at meat inspection and showed that cases tend to aggregate within specific individual farms [6]. The study also found a weak association between farm characteristics and presence of cysts. However, those farms that sent an infected animal to the abattoir tended to be at higher risk of producing more infected animals. This observation is compatible with a very low baseline risk of infection and suggests that most cases are attributable to one-off events resulting in the contamination of several animals on the same farm. The conclusion is that movement history could be a useful component of a potential risk-based inspection system, in combination with information on the age and sex of the animals. The accumulation of evidence therefore, indicates a situation where a targeted approach to surveillance for *T. saginata* cysticercus in the UK and elsewhere is worth investigating for potential efficiency gains.

Building on previous studies, we simulated a risk-based meat inspection system that uses readily available and comprehensively collected data such as the movement history, age and sex of animals at slaughter. The objective of the study was to estimate the technical performance and cost-effectiveness of different meat inspection scenarios. These estimates should be of value to inform the current discussions on the future of meat inspection of cattle in the EU and potential improvements to the cost-effectiveness and efficiency of meat inspection methods to detect *T. saginata* cysticercus in cattle.

## Methods

We designed a targeted meat inspection system based on the findings in previous studies and the possibility of conducting a new hypothetical inspection method, which involved an increase in cuts to the heart and no cuts into the masseter muscles. This hypothetical method will be referred to as ‘alternative inspection’ throughout the paper and the inspection method currently used will be referred to as ‘current inspection’. Scenarios were simulated, where different proportions of animals undergo alternative or the current inspection methods, based on the risk group to which they belong. For each scenario, the technical performance and cost-effectiveness were calculated and results compared.

### Simulation modelling

**Fig. 1:**
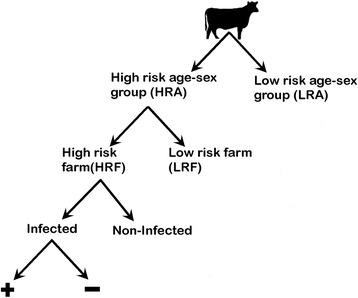
Scenario tree representation of the risk-based meat inspection system. Animals are divided into different risk categories based on the presence of high-risk farms in their movement history and the age-sex category to which they belong. Each step was assumed to be independent of others

A stochastic model (Fig. 1) with individual animals allocated to different risk groups was developed to evaluate the performance of the system under different hypothetical scenarios. The risk groups were derived from a case-control study that used inspection data from cattle slaughtered within Great Britain (GB) in the period of January 1st 2013 to January 31st 2015 [6]. Within this study, high- and low-risk farms (HRF and LRF) and high- and low-risk animals (HRA and LRA) were identified. Farms which appeared in the movement history of animals detected positive to *T. saginata* cysticercus cysts at slaughter during the period of January 1st 2013 to January 31st 2014 were identified as HRF, while only the male animals of 0–20 months of age at slaughter were identified as LRA, with all others being HRA. Hence, animals belonged to any one of the categories below:

(i) HRF and HRA: animals that have at least one high-risk farm in their movement history and are in the high-risk age-sex category;

(ii) LRF and HRA: animals that have no high-risk farms in their movement history and are in the high-risk age-sex category;

(iii) HRF and LRA: animals that have at least one high-risk farm in their movement history and are in the low-risk age-sex category;

(iv) LR or low-risk: animals that have no high-risk farms in their movement history and are in the low-risk age-sex category.

In order to account for the variability in the observed proportions of infected animals in the entire population of slaughtered animals, two stochastic processes were linked. The first was a binomial process which was used to simulate the number of infected animals (*s*) among those slaughtered (*N*):

1 $$ s= Binomial\left(N;{P}^{+}\right) $$

where *P*^*+*^ is the true prevalence of *T. saginata* cysticercus infection. Based on the result of the previous study, *P*^*+*^ = 0.086% was calculated from the apparent prevalence reported in the study and assumed sensitivity of 15% and specificity of 100% for the current inspection method using Eq. 2 [6]:

2 $$ True\ prevalence=\frac{Apparent\ prevalence+\left( Specificity-1\right)}{Specificity+\left( Sensitivity-1\right)} $$

The second was a multinomial process used to randomly allocate infected and uninfected HRA and LRA into HRF and LRF, respectively, at each iteration during simulations. The observed proportions of infected and uninfected animals within each risk category were obtained from the slaughter data for the period of January 1st 2013 to January 31st 2015 and were used to implement the stochastic process. The joint probabilities included into the multinomial process for the infected animals were calculated as follows:

3 $$ \mathrm{P}\left(\mathrm{HRF}\cap \mathrm{HRA}\right)\mid {P}^{+}=\mathrm{P}\left(\mathrm{HRF}|{P}^{+}\right)\ast \mathrm{P}\left(\mathrm{HRA}|{P}^{+}\right) $$

4 $$ \mathrm{P}\left(\mathrm{HRF}\cap \mathrm{LRA}\right)\mid {P}^{+}=\mathrm{P}\left(\mathrm{HRF}|{P}^{+}\right)\ast \mathrm{P}\left(1-\mathrm{HRF}|{P}^{+}\right) $$

5 $$ \mathrm{P}\left(\mathrm{LRF}\cap \mathrm{HRA}\right)\mid {P}^{+}=\mathrm{P}\left(1-\mathrm{HRF}|{P}^{+}\right)\ast \mathrm{P}\left(\mathrm{HRA}|{P}^{+}\right) $$

6 $$ \mathrm{P}\left(\mathrm{LRF}\cap \mathrm{LRA}\right)\mid {P}^{+}=\mathrm{P}\left(1-\mathrm{HRF}|{P}^{+}\right)\ast \mathrm{P}\left(1-\mathrm{HRF}|{P}^{+}\right) $$

where *P*(*HRF∩HRA*)*|P*^*+*^ is the estimated proportion of infected animals allocated in HRF and HRA, while *P*(*HRF|P*^*+*^) and *P*(*HRA|P*^*+*^) are the observed proportions of infected animals coming from HRF and HRA, respectively. Similarly, the joint probabilities included in the multinomial process to allocate the uninfected animals were calculated considering the observed proportions of uninfected animals belonging to each risk category.

Subsequently, animals hypothetically underwent different types of inspection procedure (i.e. alternative inspection, current inspection or no inspection) based on the risk group to which they belonged. Alternative inspection was designed based on previous studies and involved an increase in cuts into the heart (0.5–1 cm apart) and no cuts into the internal and external masseters [19, 20, 22]. The minimum, most likely and maximum values of sensitivity of the current and alternative inspection methods were adopted from a previous study conducted in Denmark which assumed the sensitivity of such an enhanced inspection to be increased by 0.1 [18]. Pert distributions were used to describe the uncertainty in sensitivity of both inspections in the model:

$$ Se= Pert\left(\mathit{\operatorname{Min}}, Most\ likely,\mathit{\operatorname{Max}}\right) $$

At inspection, the likelihood that infected animals would be detected was based on the assumed sensitivity of each inspection procedure to which they were subjected to. Model inputs, uncertainty distributions and data sources used are listed in Table 1.

**Table 1:** Values and source of inputs used in simulation modelling and cost-effectiveness analysis

Input parameter	Value	Source
• Simulation modelling		
Number of slaughtered animals	2,500,000	[6]
True prevalence of *Taenia saginata* cysticercus (*P*^*+*^)	0.00086	Calculated from apparent prevalence reported in [6], assumed sensitivity of 15% and specificity of 100%
Sensitivity of current inspection	Min = 0.04, most likely = 0.15, max = 0.25	[18]
Sensitivity of alternative inspection	Min = 0.14, most likely = 0.25, max = 0.35	[18, 20, 22]
Probability that an infected animal has high-risk farm in movement history P(HRF|P^+^)	0.45	Data collected
Probability that a non- infected animal has high-risk farm in movement history P(HRF|P^-^)	0.15	Data collected
Probability that an infected animal is a high-risk animal P(HRA|P^+^)	0.92	Data collected
Probability that a non-infected animal is from high-risk group P(HRA|P^-^)	0.78	Data collected
• Cost-effectiveness analysis		
Time taken for current full carcass inspection	3 min	Expert opinion
Time taken for alternative inspection	3.25 min	Expert opinion
Cost of meat inspectors time (per min)	£0.493	Expert opinion
Cold storing localised infected carcasses	£7	Expert opinion
Reduction in value of localised infected carcasses due to being subjected to freezing	£600	Expert opinion
Discarding of heart (i.e. heart value lost)	£1	Expert opinion
Discarding of external cheek muscle	£2	Expert opinion
Discarding of internal cheek muscle	£1	Expert opinion
Discarding of oesophagus	£1	Expert opinion
Discarding of diaphragm	£0.5	Expert opinion
Time spent removing heart	0.08 min	Expert opinion
Time spent removing external cheek muscle	0.33 min	Expert opinion
Time spent removing internal cheek muscle	0.33 min	Expert opinion
Time spent removing oesophagus	0.33 min	Expert opinion
Time spent removing diaphragm	0.25 min	Expert opinion
Decrease in value of heart due to cuts for current inspection	£1	Expert opinion
Decrease in value of external cheek muscles due to cuts for current inspection	£0.51	Expert opinion
Decrease in value of internal cheek muscles due to cuts for current inspection	£0.33	Expert opinion
Decrease in value of heart due to increased cuts for enhanced inspection	£1	Expert opinion
Carcass value (lost due to generalised infection)	£1200	Expert opinion
Disposal of carcass (discarded due to generalised infection)	£100	Expert opinion

The model was created in Microsoft® Excel and simulated using @Risk version 7 (Palisade, 2014) and was iterated 10,000 times.

The outputs from the simulations were the number of infected carcasses detected as such, the number of infected carcasses undetected, the total number of inspections (current and alternative) carried out and the number of inspections needed to find one infected carcass. To assess and quantify the impact of the uncertainty in the sensitivity of the inspection methods, results of three sub-scenarios were computed and compared. In the first, the sensitivity of both the inspection methods was kept fix to the median value of the Pert distribution; in the second and the third, the values at the 5th and 95th percentiles were used to represent the best and the worst sub-scenario, respectively.

### Validation of key model inputs

The probabilities of a bovine presented at slaughter having a “high-risk farm” in its movement history or being in the high-risk age-sex category, conditional to the infection status of the animal, were critical inputs in the simulation. To validate these inputs, an analogous study was carried out using inspection data for cattle slaughtered within the period of February-July 2015. Briefly, for each case of *T. saginata* cysticercus infection identified at slaughter during this period (*n* = 97) three controls, matched on slaughterhouse and slaughter date, were randomly selected from among the animals found to be uninfected. Age and sex data obtained from the slaughterhouse records, combined with movement history data from the British Cattle Movement Service, were used to calculate the proportions of bovines from high-risk farms and in high-risk sex-age groups, among those animals found infected and non-infected, at meat inspection. The probability of infected animals coming from these high-risk farms and the probability of each infected animal belonging to a high-risk sex-age group were calculated and compared with the inputs used in the model. The analysis was done for farms classified using only the most recent data covering the period between the 1st January 2014 and the 31st January 2015. After the high-risk farms had been defined, the number of case animals with high-risk farms in their movement history was compared to the number of controls in the six months of 2015 data.

### Expert elicitation

The opinions of eight experienced meat inspectors were elicited using a two step modified Delphi approach [23]. These meat inspectors each had at least 10 years of experience in the meat inspection of cattle in the UK had thus far inspected over 100,000 cattle in total. Inspectors were initially presented with an electronic questionnaire containing a number of scenarios designed to gather their individual views. These scenarios gleaned their opinions on the feasibility of implementing the inspection methods we had modelled. The information collected also included predicted changes in staff time associated with suggested modifications to the inspection procedure. Following an audio-visual presentation and group discussion of the collective answers to this initial questionnaire, the experts were asked to complete a secondary electronic questionnaire. The majority of this secondary questionnaire posed appropriately modified versions of previous questions, aimed at detecting a specific consensus of opinion. The results of this final secondary questionnaire were then summarised and used as inputs for the economic analysis.

### Economic analysis

**Fig. 2:**
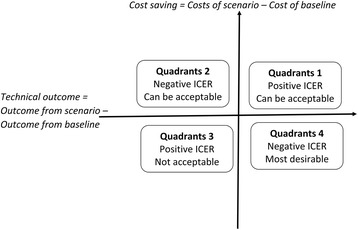
Key to the interpretation of incremental cost-effectiveness ratio (ICER) plots. ICERs with a positive value can fall within either quadrant 1 or 3. Surveillance scenarios in quadrant 1 can be acceptable in terms of cost-effectiveness, if they are within a threshold of acceptability (to be decided by the policy makers). This figure is adopted from Wall et al. [24]

The cost-effectiveness of each simulated scenario was assessed by calculating its incremental cost-effectiveness ratio (ICER) as:

7 $$ ICER=\frac{Ca- Cb}{Oa- Ob} $$

where Ca is the cost of the scenario, Cb is the cost of the baseline scenario, Oa is the technical outcome in the scenario and Ob is the technical outcome in the baseline. The interpretation of the ICER plots is presented in Fig. 2 which is adopted from a previous study [24].

The estimates of inputs used in the analysis came from the expert elicitation. It was assumed that all slaughterhouses in GB will be able to adopt all the scenarios of inspection modelled. The costs of designing the database and other changes required to conduct alternative inspection were not included in the analysis. The analysis was conducted for a year. The component costs of meat inspection included in the analysis are: meat inspector’s pay, treatment costs of locally infected carcass and decrease in value due to treatment, cost of whole carcasses disposed due to generalised infection, costs of organs and muscles disposed due to localised infection, value of carcass lost due to incising during current inspection or enhanced inspection and disposal charges.

All inputs used in the cost-effectiveness analysis were fixed values (Table 2). The economic analysis was done in Microsoft® Excel 2010.

**Table 2:** Outcomes from simulation modelling and economic analysis for the current situation and different scenarios simulated

Outcomes	Baseline All animals undergo current inspection	Scenario A HRF&HRA: Enhanced LRF&HRA: Normal HRF&LRA: Enhanced LR: no inspection	Scenario B HRF&HRA: Enhanced LRF&HRA: Normal HRF&LRA: Normal LR: Normal	Scenario C HRF&HRA: Enhanced LRF&HRA: Normal HRF&LRA: Enhanced LR: Normal	Scenario D Enhanced inspection in all animals
Total number of infected carcasses	2354 (1645–4247)	2354 (1645–4247)	2354 (1645–4247)	2354 (1645–4247)	2354 (1645–4247)
Number of infected carcasses detected	348 (336–360)	438 (338–668)	445 (352–656)	454 (353–690)	583 (361–1091)
Percent of infected carcasses detected	15 (8–21)	19 (14–23)	19 (14–24)	19 (15–24)	25 (18–31)
Number of inspections needed to find one infected carcass	7183 (6944–7440)	4630 (3050–5997)	5605 (3822–7082)	5494 (3665–7082)	4288 (2302–6887)
Number of normal inspections	2,500,000	1,657,096	2,206,747	2,123,729	0
Number of enhanced inspections	–	375,768	293,253	376,271	2,500,000
Number of animals not inspected	0	467,136	0	0	0
Total costs in million (£)	8.53 (8.52–8.54)	7.08 (7.02–7.24)	8.63 (8.57–8.77)	8.64 (8.58–8.79)	8.99 (8.84–9.33)
X = Cost of scenario – Cost of baseline	–	-1.44 (-1.51– -1.29)	0.10 (0.04–0.23)	0.11 (0.06–0.25)	0.46 (0.32–0.79)
Y = Outcome of scenario – Outcome of baseline	–	92 (2–319)	98 (6–307)	107 (16–334)	237 (25–743)
ICER = X/Y (in million £ per carcass detected)	–	-0.013 (-0.093– -0.069)	0.001 (0.0007–0.003)	0.001 (0.0007–0.003)	0.002 (0.0009–0.008)

*Abbreviations*: HRF/LRF, animals with/without a history of high-risk farms in their movement respectively; HRA/LRA, animals belonging/not belonging to high risk age-sex category, respectively; LR, low risk animals

Baseline represents current situation. Median values and 95% confidence intervals of the outcomes are presentedBaseline represents current situation. Median values and 95% confidence intervals of the outcomes are presented

## Results

### Simulation of inspection performance

The results from simulation modelling are presented in Table 2. The total number of infected carcasses a year was 2354 out of 2.5 million animals slaughtered. Current situation (baseline scenario) had the lowest number of infected carcasses detected. As a result of the low sensitivity of current inspection, in the baseline scenario, 85% of positive carcasses are missed and a total of 7183 inspections are needed to identify one positive carcass. Scenario D, where all animals undergo alternative inspection, detected the highest number of infected carcasses (based upon an assumed sensitivity of 25%).

The remaining scenarios achieve a lower number of inspections needed to identify one positive carcass.

### Validation of key model inputs

The analogous study carried out using slaughter data for GB from February 2015 to July 2015 was used to validate the probabilities used in the model. Using these 6 months of data, it was found that the probability that an infected animal had high-risk farm in movement history (P(HRF|P^+^)) was 0.34, and the probability that a non-infected animal had high-risk farm in movement history (P(HRF|P^-^)) was 0.11. In the case of age-sex category, the probability that an infected animal is a high-risk animal P(HRA|P+) was 0.94 and the probability that a non-infected animal is from a high-risk group P(HRA|P-) was 0.90.

### Expert elicitation

There was a good agreement internally between the meat inspection experts on the feasibility of the scenarios simulated. Almost 50% of the experts said the scenarios were feasible, in their opinion, for implementation in real abattoirs; 37.5% of them said the scenarios would be feasible with changes such as alterations in the line speed in high-throughput abattoirs, or with changes to staffing. The inputs for economic analysis collected through expert elicitation are presented in Table 1.

### Economic analysis

The performance, cost and ICER of the simulated scenarios are presented in Table 2. It was estimated that approximately 50% of the costs associated with the inspection are due to damage to the masseters muscles and the heart because of slicing during meat inspection. This was followed by the cost of meat inspectors’ time and by the reduction in value of the carcass due to freezing.

One of the scenarios, scenario A was less costly than the baseline. Scenario A, where all animals with high-risk farms in their movement history undergo alternative inspection; animals from the high-risk age-sex category undergo current inspection and the remaining animals undergo no inspection at all, was found to be the most cost-effective of all the scenarios tested. None of the scenarios modelled came under quadrant 3 of ICER plot which is not acceptable quadrant due to higher costs and worse outcomes.

The dominant scenario, scenario A has a median ICER of -£1300 per positive carcass detected suggesting a saving of £1300 per positive carcass detected. Scenarios B and C would cost the system £1000 per positive carcass detected and scenario D would cost £2000 per positive carcass detected.

**Fig. 3:**
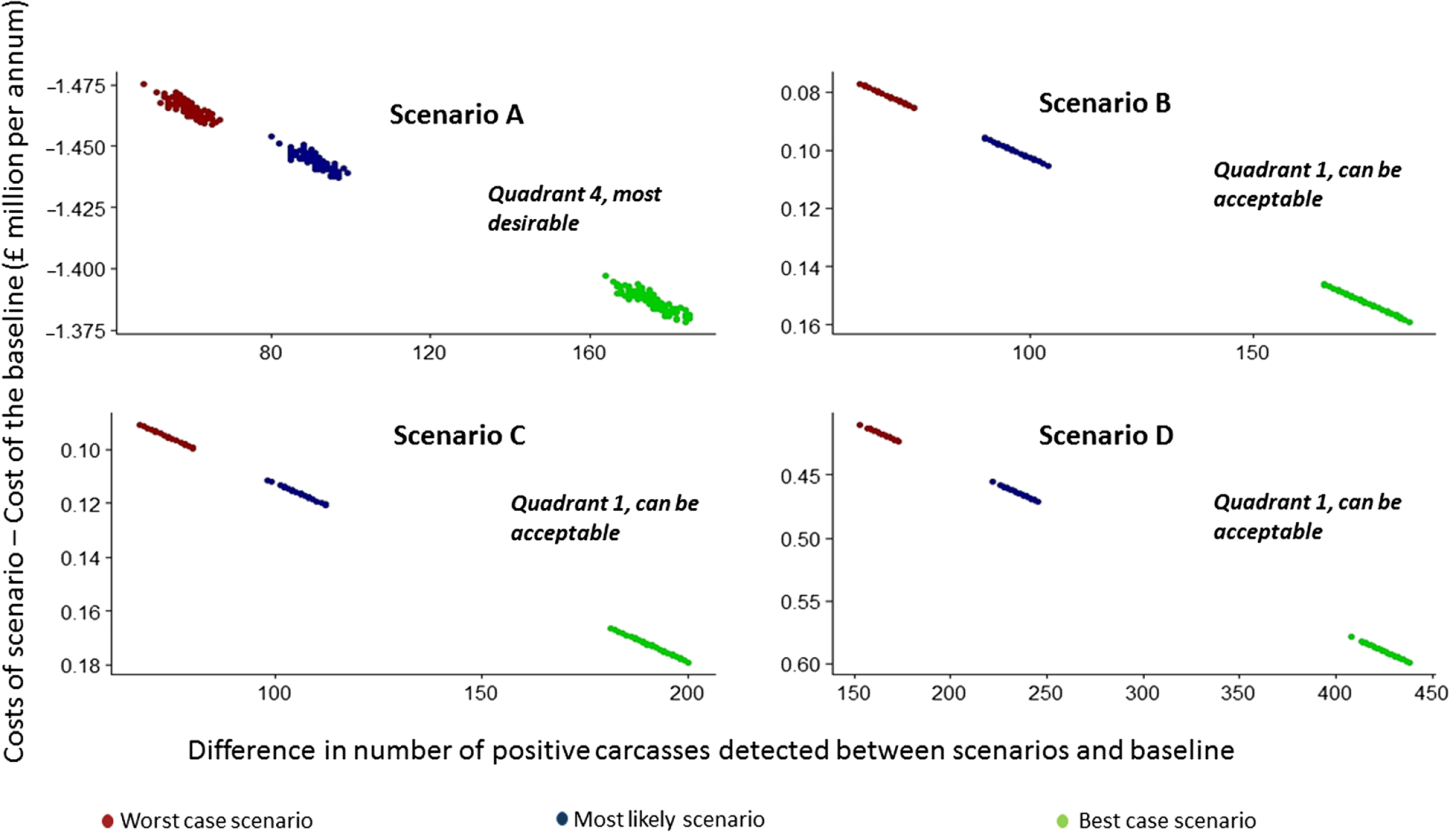
Performance of different scenarios simulated at different sensitivity values. Figure shows the performance of different scenarios simulated using 5th percentile/worst case scenario (red dots), median/most likely scenario (blue dots) and 95th percentile/best case scenario (green dots) of the sensitivity distributions. Presented here are the costs saved in each scenario *versus* the difference in the number of positive carcasses detected, compared to the baseline scenario with their interpretation based on the incremental cost-effectiveness ratio (ICER) plot. In each scenario, different proportions of animals undergo the different meat inspection methodologies, i.e. “alternative” inspection, current inspection or no inspection

**Fig. 4:**
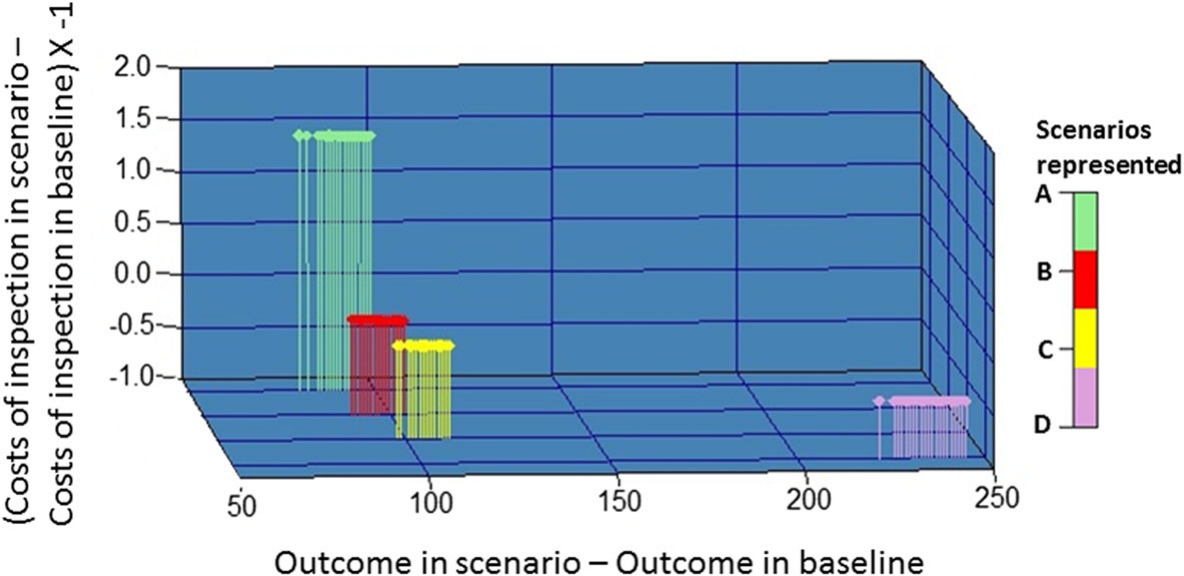
Graph comparing different scenarios when median of the sensitivity distributions are used in the simulation. *Legend*: Scenario A: HRF&HRA (represents high risk age-sex category and has at least one high risk farms in their movement history): Enhanced, LRF&HRA (represents high risk age-sex category and has no high risk farms in their movement history): Normal, HRF&LRA (HRF and LRA - represents low risk age-sex category and has high risk farms in their movement history): Enhanced and LR (low risk animals representing animals belonging to low risk age-sex category and has no high risk farms in movement history): no inspection Scenario B: HRF&HRA: Enhanced, LRF&HRA: Normal, HRF&LRA: Normal, LR: Normal. Scenario C: HRF&HRA:Enhanced, LRF&HRA: Normal, HRF&LRA: Enhanced, LRA: Normal. Scenario D: Enhanced inspection in all animals

Figure 3 represents the performance of scenarios when the best, worst and most likely values for the sensitivity of meat inspection methods were used and shows scenario A is the most desirable based on costs saved per positive carcass detected. Figure 4, where simulation results of scenarios with median values for the sensitivity are plotted, shows how the performance of scenarios compares with the baseline. It demonstrates that all the simulated scenarios perform better compared to the baseline.

## Discussion

The recent guidelines published by Codex Alimentarius recommend utilising the traceability of farms and reduced animal movements as potential control tools for *T. saginata* cysticercus, which are in line with the system modelled in this study [16]. However, it is not always possible to precisely identify a single farm as a source of infection, as an animal may have more than one farm in its movement history. Additionally, an animal may present with both viable and non-viable cysts at inspection, making it difficult to define an estimated time-point for infection [15]. With these caveats in mind, in this study we combined findings on risk factors for *T. saginata* cysticercus in GB and some EU countries to design a risk-based system that incorporates an alternative inspection, which could equate to a hypothetical alternative inspection to identify and handle carcasses infected with *T. saginata* cysticercus. In order to compare different scenarios with the baseline, we used ICERs which are commonly used in the health care industry but rarely in veterinary research except for one study on BSE [24]. The key elements of the system modelled are (i) the ability to identify carcasses with higher risk of infection at time of inspection and (ii) the ability to implement in some carcasses an alternative inspection that would result in higher probability of infected carcasses being detected.

With regards to the first element, the alternative system would utilise the movement history, age and sex of animals to identify animals at higher risk of infection as Food Chain Information which has to be received by the abattoir prior to the slaughter of the animals. Such data on individual animals are readily available as recorded information in GB and therefore could potentially be used to routinely categorize animals presented at slaughter. A critical input in the model is the strength of association between these factors and the risk of infection. This determines the conditional probabilities of infection, given the risk profile of an individual animal. Our estimates have been obtained from a two-year study in GB; different inputs would alter the technical outputs and cost-effectiveness of the scenarios. However, the analysis of data from a further six-month period revealed similar findings, which are in agreement with those of studies in other countries where there was an association between age, sex and infection status [17, 18]. The time window used to define a farm as “high-risk” is likely to modify the strength of association and this should be considered and monitored if a system such as the one we have assessed is implemented.

As for the second element, our findings depend on the ability to implement an alternative form of meat inspection, which could result in a higher sensitivity. We suggest an increase in the number of cuts to the bovine heart, to increase the sensitivity of cyst detection. During our expert elicitation there was a good consensus on incising the heart to increase sensitivity; however, there was only a negligible improvement reported in one of the studies [19]. Seven out of eight experts also agreed that the suggested changes are feasible. Furthermore, there are studies that suggest the heart as an ideal target for detailed meat inspection in countries with low prevalence [20, 22]. In the early 1990s, EU-regulated changes to meat inspection were introduced to save cost. These included reducing the number of cuts into organs such as the heart [7]. However, the heart tends to have the highest cyst density [25]. Cysts also appear to be short-lived in the heart [22], leading to faster calcification, which makes them easier to detect at meat inspection, although a study in France showed that proportion of cysticerci detected in heart using current inspection decreased with age. In this study, we assumed that it would be same for all ages. Considering the combined evidence, together with the lower economic value of the heart compared to the masseter muscles, the heart appears to be an ideal target for more detailed inspection.

As expected and in agreement with the findings of previous studies [18], the ability to apply both of these elements, targeting inspections at high-risk animals and utilising an alternative inspection with higher sensitivity can result in: (i) a scenario where costs are reduced (Scenario A); (ii) scenarios where the number of infected carcasses detected is increased (Scenarios A, B, C and D); and (iii) a scenario where costs are reduced whilst simultaneously the number of infected carcasses detected increases (Scenario A).

Scenario A, where all animals with high-risk farms in their movement history undergo enhanced inspection, those within a high-risk age-sex category undergo normal inspection, and no inspection is carried out in the remaining animals, was less costly compared to the baseline. The only dominant scenario resulting in both cost savings and improved technical efficiency was Scenario A. Under this scenario, the number of inspections required to identify an infected carcass was nearly halved with respect to the baseline scenario and it was cost-effective even in the worst case scenario studied (Fig. 3). The number of inspections needed to find one infected carcass was lower in all the scenarios tested compared to the baseline. Scenario A, B and C detected the same proportion of infected carcasses but A was less costly than B and C due to the lower number of inspections required. Approximately half a million animals are inspected in Scenario A for *T. saginata* cysticercus*.* All the scenarios studied either resulted in saved costs or detected more carcasses or did both. All the scenarios, other than Scenario A, were slightly more expensive than the baseline (Fig. 4). However, they all detected more infected carcasses.

In addition to the benefits mentioned above, a lower number of inspections and hence less incision of tissues, would not only reduce the cost of inspection but could also the chance of microbial contamination due to a reduction in the handling of muscles and organs at meat inspection. The most important meat-borne hazards today are *Campylobacter*, *Salmonella*, *Yersinia* and verotoxigenic *Escherichia coli* which can be introduced during excessive handling of muscles and cannot be detected using the traditional palpation-incision method [26]. Not conducting palpation and incision to look for *T. saginata* cysticercus cysts in half a million animals (Scenario A) may have a positive public health impact due to reduction in cross-contamination; the resources thus freed could be used elsewhere to protect public health. Furthermore, the value of muscles such as masseters will be increased in low-risk carcasses where there is no inspection for *T. saginata* cysticercus, since the whole-sale value of the muscle in its entirety is higher than where the muscle has been damaged through inspection.

In order to implement such a system, there should be an electronic database with all the high-risk farms. The data required to create a surveillance system similar to what we have modelled in this study are currently either readily available (age-sex data) or comprehensively collected (movement history), hence the costs of data collection have not been included in this assessment, as we believe the costs are negligible. Additionally, the proposed system would require regular updates, primarily regarding the identification of new potential high-risk farms. The identification of new farms where *T. saginata* cysticercus infections occur is a parallel objective of the system that we have not quantified in this study. Scenario A, where the animals from low risk farms are not subject to inspection may not fulfil this objective. This is because such a system would not allow the identification of heretofore unidentified high-risk farms and thus would not be sustainable in the long term. However, other scenarios modelled would fulfil this additional requirement, as all the animals are inspected.

This study is based on diagnoses made at meat inspection. Due to the low sensitivity of this diagnostic measure, prevalence values generated solely through this source are likely to be an underestimate. However, the uncertainty around sensitivity of meat inspection methods was incorporated and the best and worst scenarios explored. There are studies suggesting sensitivity could be lower than the most likely value included in the study (15%), hence we have included 10% as the minimum value in the stochastic model [18]. This is a good starting point and currently, there are no other adequate data sources available on prevalence of *T. saginata* cysticercus in most European countries, as pointed out in another publication [4]. A further limitation exists in that we have not considered the variability around the input costs of meat inspection. The decision to use fixed values for input costs was based on the assumption that the costs we used in the analysis are not highly variable in near future.

In the UK, the costs associated with meat inspection are distributed between farmers, abattoirs and the government. However, when a carcass is found to have localised *T. saginata* cysticercus cysts the farmer is not paid the full value of the carcass. In the case of a generalised infection, the farmer is not paid any money but he still must pay the transport and haulier charges. Hence, when a system with increased detection of infected carcasses and less or more total costs is employed, one should decide how to distribute the savings or extra costs across all stakeholders. Furthermore, the consequential costs of the potential changes (e.g. altering line speed) needed to implement such a system should be studied through stakeholder discussions and more detailed feasibility assessments.

Recently there are discussions in order to modernise the current meat inspection system for cattle as it is widely recognised within the EU that it is not well-suited to protect the public from modern microbial meat-borne hazards [26–28]. The current meat inspection system was implemented in the early 1990s, the main purposes of which are public health protection and disease surveillance. It is evident that the current inspection method for *T. saginata* cysticercus does not optimally achieve either of these objectives. Incorporating a risk-based regime cannot only detect a high number of cases, hence achieving better public health protection, but also save costs depending upon the chosen regime. In fact, some countries have already implemented risk-based meat inspection for low prevalence parasitic diseases [21, 22]. In this paper, we report only four scenarios; however, there are several other feasible options possible such as conducting current inspection only in high-risk animals, which has the potential to reduce costs considerably.

## Conclusions

A risk-based inspection regime using readily available data in the UK such as the movement history, age and sex of cattle slaughtered could improve the sensitivity of meat inspection while at the same time save money. In order to further increase the cost-effectiveness a more sensitive meat inspection method could be used on high-risk animals. Such targeting of high-risk animals would lead to fewer necessary meat inspections and less handling and degrading of tissues in the beef carcass. In turn, this should lead to lower costs to the beef industry and lower microbial contamination of beef products, improving public health outcomes. However, targeted inspection on animals from high-risk farms should be accompanied by inspection of high-risk animals from low-risk farms to ensure newly infected farms are identified by the system.

## Abbreviations

ICER: Increxmental cost-effectiveness ratios
EU: European Union
EFSA: European Food Safety Authority
UK: United Kingdom
GB: Great Britain
HRF: High-risk farm
LRF: Low-risk farm
HRA: High-risk animal
LRA: Low-risk animal
LR: Low risk
BSE: Bovine spongiform encephalopathy
